# Genomic Characterization of *Salmonella* Isolates Causing Infections in Children with Sickle Cell Disease in Dakar, Senegal

**DOI:** 10.3390/microorganisms14020506

**Published:** 2026-02-21

**Authors:** Amadou Diop, Arfang Diamanka, Adja Bousso Guèye, Baïdy Dièye, El Hadji Aly Niang, Ousmane Sadio, Mouhamadou Abdoulaye Sonko, Aïssatou Ahmet Niang, Momar Ndao, Ken Dewar, Cheikh Fall, François Paillier, Yakhya Dièye

**Affiliations:** 1 Laboratoire de Bactériologie-Virologie, Centre Hospitalier National d’Enfants Albert Royer, Dakar BP 25755, Senegal; amadoudioplaba@yahoo.fr (A.D.); baidy.dieye@ucad.edu.sn (B.D.); sonkoa160@gmail.com (M.A.S.); 2 Laboratoire de Bactériologie-Virologie, Faculté de Médecine, de Pharmacie et d’Odontologie-Stomatologie, Université Cheikh Anta Diop, Dakar BP 5005, Senegal; niangaisatou@yahoo.fr; 3 Département de Biologie Végétale, Faculté des Sciences et Techniques, Université Cheikh Anta Diop, Dakar BP 5005, Senegal; arfang.diamanka@ucad.edu.sn; 4 Pôle de Microbiologie, Institut Pasteur de Dakar, 36 Avenue Pasteur, Dakar BP 220, Senegal; adjabousso.gueye@pasteur.sn (A.B.G.); aly.niang@pasteur.sn (E.H.A.N.); ousmane.sadio@pasteur.sn (O.S.); cheikh.fall@pasteur.sn (C.F.); 5 Laboratoire de Bactériologie-Virologie, Centre Hospitalier National Universitaire de Fann, Dakar BP 5035, Senegal; 6 Division of Clinical and Translational Research, McGill University Health Centre, Montreal, QC H4A 3J1, Canada; momar.ndao@mcgill.ca; 7 Infectious Diseases and Immunity in Global Health Program, Research Institute of the McGill University Health Centre, Montreal, QC H4A 3J1, Canada; 8 Department of Human Genetics, McGill University, Montreal, QC H3A 1Y2, Canada; ken.dewar@mcgill.ca; 9 McGill Centre for Microbiome Research, McGill University, Montreal, QC H3A 2B4, Canada; 10 Aalto University Startup Center, Aalto University, 02150 Espoo, Finland; francois.paillier@aalto.fi; 11 Groupe de Recherche Biotechnologies Appliquées & Bioprocédés Environnementaux (GRBA-BE), École Supérieure Polytechnique, Université Cheikh Anta Diop, Dakar BP 5085, Senegal

**Keywords:** sickle-cell disease, invasive nontyphoidal *Salmonella*, whole-genome sequencing, plasmid, *Salmonella* pathogenicity island, antimicrobial resistance, genomics

## Abstract

*Salmonella* is a major bacterial pathogen in low-income countries, where it circulates among humans, animals, and the environment. Children with sickle cell disease (SCD) are particularly vulnerable to severe *Salmonella* infections. This study aimed to characterize *Salmonella* isolates causing infections in Senegalese children with SCD. Using antimicrobial susceptibility testing, whole-genome sequencing, and bioinformatic analysis, we investigated antibiotic resistance, serovar diversity, and virulence factors on 23 isolates from SCD patients with diverse clinical infections. The isolates belonged to 12 serovars, with Enteritidis predominating (*n* = 7). Twenty-two isolates were fully susceptible to antibiotics, while one was multidrug-resistant. Eight isolates (Enteritidis and Typhimurium) carried a virulence plasmid harboring the *spvRABCD* gene cluster. Core *Salmonella* pathogenicity islands (SPIs-1 to -5, -11, and -13), as well as SPI-10 and SPI-23, were detected in all isolates, whereas other SPIs were variably present. These results show high serovar diversity and low antimicrobial resistance among *Salmonella* isolates in children with SCD in Dakar, Senegal. Our findings suggest that strains causing diarrhea in healthy individuals may also cause invasive disease in SCD patients, highlighting the need for dedicated surveillance in this vulnerable population.

## 1. Introduction

*Salmonella enterica* is a bacterium that comprises over 2600 serovars, also called serovars [1]. *S. enterica* infects various human and animal hosts orally and primarily causes two types of diseases: gastroenteritis and invasive infection. *Salmonella* gastroenteritis manifests as diarrhea, abdominal pain and vomiting and is characterized by bacterial colonization restricted to the intestines [2]. Healthy individuals typically recover after a few days. In contrast to *Salmonella* gastroenteritis, invasive salmonellosis is a life-threatening disease characterized by the translocation of bacteria to the systemic compartment of the infected host. During invasive salmonellosis, bacteria invade epithelial cells and mucosa-associated lymphoid tissue, survive within phagocytic cells and are transported to the liver and the spleen [3]. Bacteria are ultimately released from these organs into the bloodstream and disseminate widely throughout the host. The most common sites of secondary infection are the bone marrow and the gallbladder [4]. Dissemination of bacteria may result in complications including gastrointestinal perforation; hepatitis; meningitis; and cardiovascular, neurological, urinary and respiratory infections [5]. The cardiovascular complications of *Salmonella* infections are mostly intravascular infections and endocarditis [5]. Although rare, these infections are serious threats because their diagnosis is difficult and their treatment often requires surgical intervention. Furthermore, *Salmonella* endocarditis may evolve into cerebral infection [6]. A proportion of patients who have recovered from *Salmonella* infection can become healthy carriers and contribute to the dissemination of bacteria.

Invasive salmonellosis includes typhoid and Paratyphoid fever caused by the human-restricted serovars Typhi and Paratyphi and invasive nontyphoidal salmonellosis (iNTS) that is caused by serovars mostly known to be associated with *Salmonella* gastroenteritis. Typhimurium and Enteritidis are the most frequently isolated iNTS serovars [7,8]. This is of epidemiological importance given that these serovars can infect multiple hosts and are transmissible through various sources, including human, animal and environmental origins. Available data on iNTS in sub-Saharan Africa (sSA) are sparse and not aggregated, making it difficult to obtain a broad epidemiological view of the burden of this disease. A systematic review that compiled reports on iNTS in Africa from 1966-2014 reported that Enteritidis and Typhimurium accounted for 91% of the iNTS cases in which the serovar was determined [8]. This and other studies also reported that iNTS cases were frequent in coinfections, especially in HIV-infected and malaria patients. Typhoid fever and iNTS cause over 25 million cases and nearly 900,000 deaths annually worldwide, and most of these cases occur in Africa [7]. Newborn, infant, elderly and immunocompromised individuals are more susceptible to *Salmonella* infection. Among these populations, sickle-cell disease (SCD) patients are of particular concern due to their heightened susceptibility to *Salmonella* [9].

SCD is one of the most common inherited hemoglobin disorders worldwide, affecting more than 300,000 newborns each year, most of whom live in sub-Saharan Africa [10]. SCD is caused by an autosomal recessive mutation in the human β-globin gene that alters both the structure and function of hemoglobin. This disorder is associated with a wide range of complications, including severe anemia, acute and chronic pain, avascular necrosis, priapism, acute chest syndrome, stroke, splenic dysfunction, organ failure, and life-threatening infections [11]. Bacterial infections represent a leading cause of morbidity and mortality in individuals with SCD, especially children [12]. This heightened susceptibility is primarily due to functional asplenia, which commonly develops early in life due to repeated splenic infarctions caused by sickled red blood cells. The spleen plays a vital role in filtering encapsulated bacteria and initiating immune responses, and its dysfunction renders children with SCD highly vulnerable to pathogens such as *Streptococcus pneumoniae*, *Haemophilus influenzae*, and *Salmonella* [12]. The pathogenesis of these infections is aggravated by impaired macrophage function and reduced complement activity in SCD patients [13]. The ability of *Salmonella* to invade host epithelial cells, survive within macrophages, and evade immune clearance enhances its pathogenic potential in immunocompromised individuals such as those with SCD [14]. *Salmonella* pathogenicity is mediated by an array of virulence factors encoded within discrete chromosomal regions known as *Salmonella* pathogenicity islands (SPIs) [15]. To date, 24 SPIs have been identified, several of which have been extensively characterized. However, to our knowledge, the specific contribution of these SPIs to pathogenesis in individuals with SCD remains unexplored.

In Senegal, where an estimated 10–11% of the population carries the sickle cell trait, the burden of SCD is important [16]. Despite the clinical relevance of *Salmonella* infections in African children with SCD, data remain limited on the genomic characteristics of isolates causing clinical infections in this population. In this study, we aimed to characterize *Salmonella* isolates causing infections in children with SCD. Our objectives were to identify the serovars associated with both gastrointestinal and invasive diseases and to determine their antibiotic resistance determinants and virulence factors. We performed whole-genome sequence and genomic analyses of 23 *Salmonella* isolates from affected children. Here we report the serovars, STs, plasmids, as well as the pathogenicity islands and antibiotic resistance determinants of the analyzed strains.

## 2. Materials and Methods

### 2.1. Bacterial Culturing and Antimicrobial Susceptibility Testing

This study was approved by the Université Cheikh Anta Diop de Dakar’s institutional research ethics committee (Protocol No 0079/2015/CER/UCAD). We analyzed 23 archived *Salmonella* strains stored at the microbiology laboratory of the Children’s Hospital, Dakar, Senegal (Table 1). This laboratory typically discards bacterial isolates after routine investigation. Selected isolates of potential interest are stored at −80 °C in nutrient broth (Sigma Aldrich, St. Louis, MO, USA) supplemented with 10% skim milk. The strains included in this study were isolated from SCD children suffering from different infections between 2007 and 2019 (Table 1). All the stored isolates were successfully revived in Rappaport broth (Biomérieux, 69280 Marcy-l’Étoile, France) at 37 °C, then cultured on Mueller-Hinton plates (Biomérieux, 69280 Marcy-l’Étoile, France) at 37 °C, and species identity was verified on a MALDI-TOF biotyper (Bruker Daltonik GmbH, 28359 Bremen, Germany). Antimicrobial susceptibility testing (AST) was performed using the disc diffusion method on Mueller-Hinton agar plates. The plates were inoculated with a bacterial suspension equivalent to a 0.5 McFarland standard, corresponding to approximately 1–2 × 10^8^ CFU/mL. We tested a variety of antibiotics in order to identify resistance mechanisms relevant to *Enterobacterales*. The antibiotic discs (Bio-Rad Antibiotic Disks, Marnes la Coquette, France) used included ampicillin (10 µg), ticarcillin (75 µg), cefalotin (30 µg), cefoxitin (30 µg), cefotaxime (30 µg), ceftazidime (30 µg), cefepime (30 µg), imipenem (10 µg), gentamicin (10 µg), nalidixic acid (30 µg), norfloxacin (10 µg) and chloramphenicol (30 µg). The results were interpreted following the recommendations of the European Committee on Antimicrobial Susceptibility Testing (CA-SFM/EUCAST 2025 guidelines, www.sfm-microbiologie.org, accessed on 19 January 2026) and were recorded as susceptible, intermediate or resistant. Multidrug resistant (MDR) bacteria were defined as isolates resistant to at least one molecule of at least three antibiotic families. Strain *E. coli* ATCC 25922 was used for AST quality control.

### 2.2. Extraction of Bacterial Genomic DNA, Whole-Genome Sequencing and Genome Assembly

Bacterial genomic DNA was extracted using a Qiagen DNA Mini Blood & Tissue Kit (Qiagen, Germantown, MA, USA) according to the manufacturer’s recommendations. The quality and concentration of the extracted DNA were determined with a NanoDrop^TM^ 2000/2000c spectrophotometer (Thermo Fisher Scientific, Waltham, MA, USA) and a Qubit 3.0 fluorometer (Thermo Fisher Scientific). For Illumina sequencing, DNA libraries were prepared with the Nextera XT DNA Library Preparation Kit (Illumina, San Diego, CA, USA) following the manufacturer’s instructions. The resulting DNA libraries were purified via AMPure XP beads (Beckman Coulter, Sharon Hill, PA, USA) and quantified with a Qubit 3.0 fluorometer (Thermo Fisher Scientific) to obtain a normalized library pool. Sequencing was performed on a MiSeq platform with v2 sequencing reagent kits (Illumina). The quality of the sequencing reads was evaluated with FastQC v0.11.9 (https://www.bioinformatics.babraham.ac.uk/projects/fastqc/, accessed on 19 January 2026) before trimming with Trimmomatic v0.39 (https://trimmomatic.com/, accessed on 19 January 2026). Reads that passed quality control were submitted to mash v1.1 (https://mash.readthedocs.io/en/latest/, accessed on 19 January 2026) to determine the similarity of our isolates to genomes in the NCBI Refseq database and to select the genetically closest strain to be used for reference-guided assembly. Reads that did not map to the reference chromosome were considered to form a putative plasmid and were assembled *de novo* with SPAdes v3.15.3 (https://github.com/ablab/spades, accessed on 19 January 2026). The assembled contigs were queried against the PLSDB plasmid database (https://www.ccb.uni-saarland.de/plsdb/, accessed on 19 January 2026) via local BLAST v2.10.

### 2.3. Serovar Prediction, Detection of Antimicrobial Resistance and Virulence Genes, and Phylogeny

*Salmonella* serovars were predicted by submitting the contigs from the genome assembly to the SeqSero 2 (http://denglab.info/SeqSero2, accessed on 19 January 2026) platform. Sequence types were determined via multilocus sequence typing (MLST) 2.0 software. The detection of antimicrobial resistance genes (ARGs), virulence factors, and plasmid replicons was performed via Abricate v1.0.1 (https://github.com/tseemann/abricate, accessed on 19 January 2026), which screens assemblies against multiple curated databases, including ResFinder, CARD, NCBI, and VFDB. To investigate the presence of *Salmonella* Pathogenicity Islands (SPIs), a custom local database comprising SPI-1 to SPI-21 sequences from previously published references was created [15,17], along with SPI-22, SPI-23, and SPI-24 retrieved from GenBank (accession numbers: FR877557.1 nucleotides 1,249,885–1,369,393; LAZB01000005.1 nucleotides 1,159,333–1,196,365; and AF140550, respectively). BLAST searches were used to compare assembled genomes against this database, with a minimum threshold of 60% sequence coverage and 90% nucleotide identity required to confirm the presence of a given SPI.

Multilevel genome typing (MGT) was performed by submitting sequencing reads from the seven Enteritidis isolates to the MGT database maintained at the University of New South Wales, Sydney, Australia (MGTdb, https://mgtdb.unsw.edu.au/enteritidis/, accessed on 19 January 2026). Existing alleles for each level were automatically assigned, and new allele numbers were requested for alleles not present in the database. Phylogenetic analysis was performed via the EnteroBase web platform (http://enterobase.warwick.ac.uk/species/index/senterica, accessed on 19 January 2026). A total of 193 *Salmonella enterica* serovar Enteritidis isolates from different continents were selected, with roughly equal numbers of isolates per continent, including seven isolates from this study. An SNP-based phylogenetic tree was generated via EnteroBase’s built-in pipeline and constructed with RAxML via the maximum likelihood algorithm (https://enterobase.readthedocs.io/en/latest/pipelines/enterobase-pipelines.html#snp-trees, accessed on 19 January 2026). The tree was refined for visualization in RStudio (v2024.12.1) with the packages ggplot2 and ggtree.

### 2.4. Sequencing Data Availability

The raw Illumina sequencing data (FastQ files for paired-end reads) were uploaded to the EnteroBase web-based platform (http://enterobase.warwick.ac.uk/species/index/senterica, accessed on 19 January 2026) and analyzed via its available tools to confirm the read quality, isolate serovars, MLST, and detection of virulence and antimicrobial resistance genes. These data are available at the EnteroBase platform. The assembled genomes are available at the National Center for Biotechnology Information (NCBI) under the Bioproject numbers PRJNA1289010, PRJNA1289517, PRJNA1394221. The accession numbers of the isolates can be found in Supplementary Table S1. All the other relevant data related to this study are contained in this manuscript.

## 3. Results

We performed whole-genome sequencing (WGS) of 23 *Salmonella* isolates stored at the microbiology laboratory of the Children’s Hospital in Dakar, Senegal. The isolates were recovered from stool (*n* = 9), urine (*n* = 5), blood (*n* = 5), cerebrospinal fluid (*n* = 2), lung aspirate (*n* = 1), and ascites (*n* = 1) samples from children with SCD (Table 1). Since *Salmonella* infection is frequent in SCD patients and since this bacterium is well spread in low- and middle-income countries (LMICs), we wanted to verify the serovar diversity and further characterize the isolates. For this purpose, we performed antimicrobial susceptibility testing and genomic analysis of the strains.

### 3.1. Serovar Diversity of Salmonella Isolates from Children with Sickle-Cell Disease

SeqSero analysis of the WGS data revealed 12 serovars, with Enteritidis (seven isolates) being predominant, followed by Chester (five isolates) and Typhimurium (two isolates), whereas one isolate was found for serovars Albany, Brandenburg, Bredeney, Colindale, Johannesburg, Lille, Liverpool, Typhi and Virchow (Table 1). These results show that a variety of *Salmonella* serovars infect SCD patients, which is consistent with a previous study where we reported 16 different serovars among 19 stool samples from individuals suffering from diarrhea [18]. The predominance of Enteritidis was also consistent with this serovar being associated with both diarrheal and invasive *Salmonella* infection in sub-Saharan Africa [7,8]. Notably, eight of the 12 serovars were recovered from extraintestinal samples, showing that these bacteria could cause invasive disease in SCD patients known to be susceptible to *Salmonella* infection [19]. MLST analysis revealed that the 23 isolates belonged to known STs and that all the strains belonging to the same serovars shared a single ST, including ST11, ST1954 and ST19 for Enteritidis, Chester and Typhimurium, respectively (Table 1).

### 3.2. Antimicrobial Resistance of Salmonella from Children with Sickle-Cell Disease

Antimicrobial susceptibility testing revealed that all but one isolate was susceptible to all the antibiotics tested. The isolate displaying resistance belonged to the Enteritidis serovar and was a multidrug-resistant (MDR) strain resistant to penicillin, tetracycline, chloramphenicol, and anti-folate families of antibiotics (Table 2). The presence of antibiotic resistance genes was consistent with the resistance profile of this isolate, with *bla_TEM_* (penicillin resistance), *catA1* (chloramphenicol resistance), *dfrA17* (trimethoprim resistance), *sul1* (sulfonamide resistance), *sul2* (sulfonamide resistance), and *tetB* (tetracycline resistance) genes identified in addition to *aph(6)-Id* and *aph(3”)-Ib*, two aminoglycoside-phosphatidyl transferases that encode resistance to aminoglycosides, an antibiotic class to which *Salmonella* is intrinsically resistant (Table 2).

### 3.3. Plasmid Profiling in Salmonella from Children with Sickle-Cell Disease

Among the 23 isolates analyzed, 15 harbored a plasmid, including all seven Enteritidis strains; four of five Chester strains; one of the two Typhimurium strains; and the Brandenburg, Johannesburg, and Liverpool isolates (Table 1). It is worth noting that our detection method may have missed cryptic plasmids, particularly small ones. Analysis of the Enteritidis and Typhimurium isolates revealed the co-occurrence of IncFIB(S) and IncFII(S) replicons on the same plasmid backbone, which is consistent with previously described multireplicons in *Salmonella* [20]. These plasmids were ~59 kb and 93.9 kb long in the Enteritidis and Typhimurium isolates, respectively (Table 1), and encoded known virulence factors associated with the *Salmonella* virulence plasmid. These genes included the *spvRABCD* virulence genes essential for systemic infection; the *pefABCD* (plasmid-encoded fimbriae) operon, which mediates adhesion to the intestinal epithelium; and the *rck* gene, which enables resistance to complement killing, cell invasion and survival. TW11, the remaining Enteritidis isolate, harbored a 67.8 kb IncI1-1α plasmid that encoded the *spvRABCD* virulence genes. Additionally, this plasmid contained *bla_TEM_*, a class A β-lactamase. A BLAST search of the pTW11 sequence against the NCBI database retrieved one plasmid of identical size (67.8 kb) from an Enteritidis isolate originating from sub-Saharan Africa, showing 99.9% nucleotide identity (accession number LR794377). In addition, numerous plasmids of approximately 59 kb partially matched the pTW11 sequence. These findings suggest that pTW11 likely originated from the 59 kb Enteritidis virulence plasmid through the acquisition of additional genetic material or via fusion with another plasmid. The other plasmids found in the isolates did not contain known virulence or antibiotic resistance genes. These include large 113 kb incFIB(pB171) and 92 kb IncFII(S) replicons in the Brandenburg and Johannesburg strains, respectively, and small Col-type plasmids (3.6–4 kb) Col_156 present in four Chester isolates and Col(MGD2) in the Liverpool isolates (Table 1).

### 3.4. Salmonella Pathogenicity Islands in Isolates from Children with Sickle-Cell Disease

**Figure 1:**
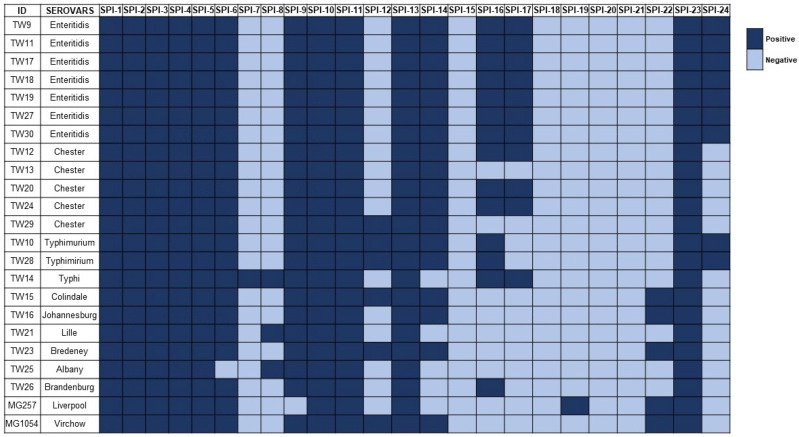
Heatmap of *Salmonella* Pathogenicity Island distribution among isolates from children with sickle cell disease. Assembled genomes of the isolates were analyzed using BLAST searches against a custom local database containing 24 known *Salmonella* Pathogenicity Islands (SPIs). The presence of each SPI was confirmed based on a minimum threshold of 60% sequence coverage and 90% nucleotide identity.

We analyzed the 23 isolates for the presence of 24 known SPIs. As expected, SPI-1 through SPI-5, SPI-11 and SPI-13, reportedly present in all *Salmonella* serovars, were detected in every isolate (Figure 1). SPI-10 and SPI-23 were also universally present. SPI-6 and SPI-9, though also ubiquitous in *Salmonella* serovars, were absent in one isolate each. In contrast, other SPIs described as ubiquitous in *Salmonella enterica* subspecies *enterica* serovars showed variable distribution. SPI-14 was found in 18 strains, SPI-24 was present by nine strains (exclusively Enteritidis and Typhimurium), and SPI-12 was present in six isolates (Figure 1). Further analysis revealed SPI-16 in 14 and SPI-17 in 11 isolates, with the latter present in all the Enteritidis (*n* = 7), Typhi (*n* = 1) and three Chester isolates. SPI-22 was present in five strains (Figure 1). Notably, SPI-8, described as specific to the serovars Typhi and Paratyphi, was also found in the Albany and Lille isolates. SPI-7 and SPI-19 were each detected in a single isolate (Typhi and Liverpool, respectively). No isolates met the detection criteria (≥60% coverage and ≥90% identity) for SPIs 15, 18, 20 and 21. Overall, SPI profiles were largely conserved within serovars, and no specificity was observed correlating with infection type (gastrointestinal vs. invasive). These findings are consistent with recent genomic studies analyzing SPI profiles from WGS data [15,21].

### 3.5. Multilevel Genome Typing of Enteritidis Isolates from Children with Sickle-Cell Disease

**Figure 2:**
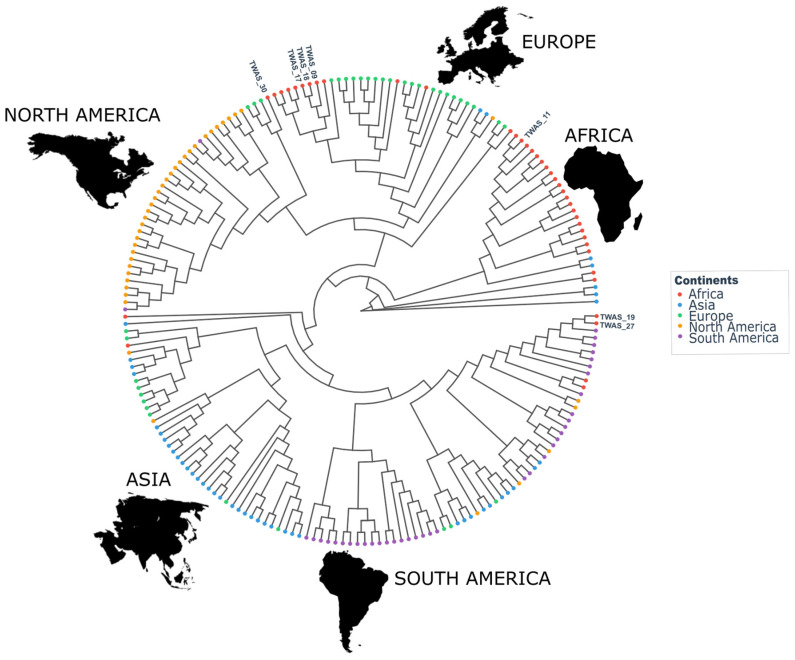
Whole-genome single-nucleotide polymorphism-based phylogenetic analysis of *Salmonella enterica* serovar Enteritidis isolates. Genomes of 193 Enteritidis isolates, including the seven generated in this study, were retrieved from the EnteroBase database platform (http://enterobase.warwick.ac.uk/species/index/senterica, accessed on 19 January 2026) and represent strains from multiple continents. An SNP-based phylogenetic tree was constructed using EnteroBase’s built-in pipeline with RAxML under the Maximum Likelihood algorithm. The resulting tree was exported and refined for visualization in RStudio version 2026.01.0 using the ggplot2 and ggtree packages. The geographical origins of the analyzed isolates were color-coded.

Since Enteritidis was predominantly found among the isolates associated with *Salmonella* diseases in sub-Saharan Africa, we wanted to further characterize our isolates belonging to this serovar. For this purpose, we submitted the corresponding WGS data to a recently developed multilevel genome typing database (MGTdb) that uses a core genome SNP analysis to determine a genome type, which corresponds to a string of nine sequence types with increasing genotypic resolution [22]. Please note that MGT analysis is available only for the *Salmonella* serovars Enteritidis and Typhimurium. Corresponding allele numbers up to level 8 were found for one isolate, TW11, the MDR strain (Table 3). Interestingly, its ST at level 4 (MGT4-ST11) was shared by an isolate predominantly present in West Africa [22]. Additionally, TW11 belongs to MGT3-ST10, which mainly includes MDR isolates [22]. In addition to TW11, the remaining isolates presented corresponding allele numbers for levels 1–6, with two strains missing one intermediary level each (Table 3). This observation is not surprising since African isolates represent less than 7% of the strains in the MGT database (https://mgtdb.unsw.edu.au/enteritidis/isolate-list, accessed on 19 January 2026) maintained at the University of New South Wales, Sydney, Australia. Interestingly, isolates TW19 and TW27, which originated from CSF and stool, respectively, shared the same six allele numbers, indicating close genetic proximity. These isolates share MGT4-ST15, a global ST found in isolates present on all continents [22]. To further analyze our Enteritidis isolates, we constructed a whole-genome SNP-based phylogenetic tree with Enteritidis strains from the Enterobacteria database. We randomly selected 193 isolates with roughly similar numbers of isolates per continent. The tree revealed continent-specific clusters as well as clades composed of isolates from different geographical origins (Figure 2). Interestingly, TW11 clustered with African isolates, which is consistent with the findings from the MGTdb analysis above (Figure 2). Similarly, in agreement with MGTdb typing, TW19 and TW27 belonged to a cluster composed of isolates from different geographical origins (Figure 2). The remaining four isolates clustered with strains of African origin (Figure 2).

## 4. Discussion

In this study, we conducted a genomic characterization of 23 *Salmonella* isolates obtained from SCD children from Dakar, Senegal. The isolates belonged to 12 *Salmonella* serovars, with Enteritidis being the most common. All but one isolate were susceptible to all the antibiotics tested, the last being an MDR strain resistant to penicillins, chloramphenicol, tetracycline and antifolates. The diversity of *Salmonella* serovars causing clinical infection in SCD patients is not surprising since a similar observation was reported in non-SCD individuals [18]. We observed comparable serovar diversity in isolates from both gastrointestinal and invasive infections. Given the limited data on serovars causing infections in individuals with SCD, our findings suggest that serovars commonly associated with gastroenteritis in healthy individuals may also drive invasive disease in this population. This observation is particularly important in LMICs, where individuals with SCD face high exposure to *Salmonella,* to which they are especially vulnerable. Indeed, *Salmonella* is widely distributed in LMICs, mainly because of poor hygiene, with asymptomatic carriage in humans [23] and a high prevalence in animals, food and the environment. This concern is further exacerbated by the continuously rising prevalence of iNTS in sub-Saharan Africa. Enteritidis, Typhimurium and other serovars [24,25], including those analyzed in this study, can infect humans and various animal hosts and can be contracted through the food chain. Indeed, food animals such as poultry, cattle and pigs and their products (e.g., milk and meat) constitute important sources of *Salmonella* contamination [26]. These naturally resistant animals serve as reservoirs of serovars that can be transmitted to humans. In addition to animals, fruits and vegetables can be important vectors of *Salmonella* contamination [27]. In a study evaluating the risk of ingesting *Salmonella* from vegetables, we reported a high prevalence of *Salmonella* present in leafy vegetables (mint, parsley, and lettuce) sold in open markets and supermarkets in the western coastal region of Senegal, an area with intense gardening activity [27]. Given their sensitivity to *Salmonella*, it is highly recommended that SCD individuals take precautions and follow strict hygienic practices, respecting disciplined alimentary habits to minimize the possibility of being infected by *Salmonella*. This is challenging in the sub-Saharan African context, where large family sizes favor person-to-person transmission. Moreover, the poor public infrastructure in LMICs enhances the spread of *Salmonella* and other pathogenic bacteria. There is a need for integrated surveillance based on a one-health approach that will help mitigate the risk represented by severe *Salmonella* infection in SCD in Senegal and other LMICs where this genetic trait is significantly present.

Vaccination is the best means to control *Salmonella* and other infections in children with SCD. There are currently three licensed vaccines against *Salmonella*. One is a live attenuated strain that requires multiple administrations of high doses to protect against serovar Typhi and Paratyphi B [28]. Because of its live bacterial status, this vaccine is not approved for children younger than 5 years or for immunodeficient individuals. The second vaccine is a conjugate of the Vi capsular polysaccharide that confers significant protection after a single administration [28]. However, this vaccine is inefficient in children younger than 2 years, and its protection is restricted to serovar Typhi. The third vaccine was approved by the World Health Organization in 2018 and has been used in India and Nepal for children aged 6 months and older [29]. However, like the other two vaccines, it only protects against serovar Typhi. In addition to these vaccines, there are ongoing development programs. However, no vaccine against NTS or suitable for immunodeficient individuals is currently available. An ideal *Salmonella* vaccine would have broad serovar coverage to protect against both gastrointestinal and systemic salmonellosis and would be safe for babies and immunocompromised individuals, including those with SCD, the most vulnerable subjects in sub-Saharan Africa. The development of such vaccines requires advanced preclinical studies. In this context, the use of animal models is relevant. There are few SCD mouse models that are used for experimental infection. These models have enabled important investigations into the pathogenesis of *Streptococcus pneumoniae*, the leading bacterial infection in individuals with SCD, and have been used to evaluate treatment [30]. Interestingly, comparative experimental infections of *S. pneumoniae* isolates from SCD and non-SCD individuals revealed differential virulence factor expression in the two bacterial populations, demonstrating that host-pathogen molecular interactions impact pathogenesis and bacterial fitness within the SCD host [30]. *Salmonella* pathogenesis has been extensively studied in a mouse model, with numerous genetic tools developed to decipher the molecular functions of numerous virulence factors [31]. However, to our knowledge, there are no reports of *Salmonella* pathogenesis in an SCD mouse model. Given the high diversity of *Salmonella* serovars infecting SCD and the knowledge accumulated on virulence factors, especially SPIs, SCD mouse models would be valuable for the study of pathogenesis, the characterization of virulence factors relevant to SCD, and the identification of targets for therapeutic intervention. Additionally, SCD mouse models can serve in preclinical studies to test candidate vaccines for iNTS and evaluate their efficacy in the context of SCD.

We acknowledge two important limitations of this study. First, the small sample size prevented a comparative analysis of serovars causing gastrointestinal versus invasive disease. Consequently, while we observed high serovar diversity, we could not determine whether specific serovars are primarily responsible for invasive salmonellosis in children with SCD. Second, our detection threshold for SPIs required ≥60% coverage and ≥90% nucleotide identity. Given the known variability in SPI content and sequences, this conservative threshold may have led to the omission of some SPIs, limiting comparative analyses.

## 5. Conclusions

This study reveals a diversity of *Salmonella* serovars associated with infections in Senegalese children with SCD, with Enteritidis being the most prevalent. Antibiotic resistance was infrequent among the isolates analyzed. Plasmid content and SPI profiles were generally conserved within serovars, and no association was found between these genetic features and infection type. Together, these findings highlight the urgent need for enhanced surveillance through active programs that incorporate genomic characterization of *Salmonella* isolates from healthy carriers, SCD patients, and other high-risk groups.

## Figures and Tables

**Table 1:** Serovar, sequence types and plasmids of *Salmonella* isolates from children with sickle-cell disease.

ID	Year of Isolation	Source	Serovars	ST	Plasmid Replicon (Size)	Plasmid Virulence Genes
TW18	2016	CSF	Enteritidis	11	IncFII(S)/IncFIB(S) (58.3 kb)	*spvRABCD* *pefABCD* *rck*
TW19	2018	CSF	Enteritidis	11	IncFII(S)/IncFIB(S) (59.4 kb)	*spvRABCD* *pefABCD* *rck*
TW09	2018	Blood	Enteritidis	11	IncFII(S)/IncFIB(S) (59.4 kb)	*spvRABCD* *pefABCD* *rck*
TW11	2010	Blood	Enteritidis	11	IncI1_1_alpha (67.9 kb)	*spvRABCD*
TW17	2016	Urine	Enteritidis	11	IncFII(S)/IncFIB(S) (58.3 kb)	*spvRABCD* *pefABCD* *rck*
TW27	2007	Stool	Enteritidis	11	IncFII(S)/IncFIB(S) (58.3 kb)	*spvRABCD* *pefABCD* *rck*
TW30	2019	Stool	Enteritidis	11	IncFII(S)/IncFIB(S) (57.2 kb)	*spvRABCD* *pefABCD* *rck*
TW12	2018	Blood	Chester	1954	Col156_1 (4.0 kb)	None
TW13	2018	Blood	Chester	1954	Col156_1 (4.0 kb)	None
TW20	2018	Lung aspirate	Chester	1954	Col156_1 (4.0 kb)	None
TW24	2010	Stool	Chester	1954	Col156_1 (4.0 kb)	None
TW29	2018	Stool	Chester	1954	None	NA
TW10	2010	Blood	Typhimurium	19	IncFII(S)/IncFIB(S) (93.9 kb)	*spvRABCD* *pefABCD* *rck*
TW28	2016	Stool	Typhimurium	19	None	N/A
TW14	2016	Urine	Typhi	2	None	N/A
TW15	2007	Urine	Colindale	584	None	N/A
TW16	2016	Urine	Johannesburg	515	IncFII(S) (92.1 kb)	None
M1054	2019	Urine	Virchow	303	None	N/A
TW21	2019	Ascites	Lille	297	None	N/A
TW23	2008	Stool	Bredeney	505	None	N/A
TW25	2010	Stool	Albany	292	None	N/A
TW26	2009	Stool	Brandenburg	65	IncFIB(pB171) (113.0 kb)	None
M257	2019	Stool	Liverpool	1959	Col(MGD2)	None

CSF, cerebrospinal fluid; Peritoneal, peritoneal fluid.

**Table 2:** Phenotypic and genotypic antibiotic resistance features of multidrug resistant Enteritidis isolate.

ID	Serovar	ST	Resistance Profile	ARG—Chromosome	ARG—Plasmid
TW11	Enteritidis	ST11	Ampicillin, Ticarcillin, Chloramphenicol, Trimethoprim–Sulfamethoxazole, Tetracycline	*catA1*, *dfrA17, sul1*, *sul2*, *tetB, aph(6)-Id* and *aph(3″)-Ib*	*bla_TEM_*

ST, Sequence Type; ARG, antibiotic resistance gene.

**Table 3:** Multilevel Genome Typing Sequence Type of Salmonella Enteritidis from children with sickle-cell disease.

ID	Source	Isolation	MG1-ST	MG2-ST	MG3-ST	MG4-ST	MG5-ST	MG6-ST	MG7-ST	MG8-ST	MG9-ST
TW09	Blood	2018	**11**	**1**	19	58	**67**	11919	X	X	X
TW19	CSF	2018	**11**	**1**	**1**	**15**	**1**	**3884**	X	X	X
TW27	Stool	2007	**11**	**1**	**1**	**15**	**1**	**3884**	X	X	X
TW18	CSF	2016	**11**	**1**	**686**	X	5841	**3516**	X	X	X
TW17	Urine	2016	**11**	**1**	**686**	58	**67**	**3516**	X	X	X
TW11	Blood	2010	**11**	5	10	11	11	12	12	31144	X
TW30	Stool	2019	**11**	271	1302	2423	X	7875	X	X	X

Cleaned raw sequencing reads were submitted to the Multilevel Genome Typing database platform (MGTdb, https://mgtdb.unsw.edu.au/enteritidis/, accessed on 19 January 2026) for allelic assignment across the nine-level MGT scheme. Numbers indicate existing sequence types (STs) at the corresponding hierarchical levels; numbers in bold indicate sequence types shared by two or more isolates at a given level. “X” denotes new allelic profiles for which sequence types have not yet been assigned within the database. MGX-ST, Multilevel Genome Type at level X—Sequence Type; CSF, cerebrospinal fluid.

## Data Availability

The raw Illumina sequencing data (FastQ files for paired-end reads) were uploaded to the EnteroBase web-based platform (http://enterobase.warwick.ac.uk/species/index/senterica, accessed on 19 January 2026). The assembled genomes were also submitted and are available at the National Center for Biotechnology Information (NCBI) under the Bioproject numbers PRJNA1289010, PRJNA1289517, PRJNA1394221. All the other relevant data related to this study are in this manuscript.

## Supplementary Materials

The following supporting information can be downloaded at: https://www.mdpi.com/article/10.3390/microorganisms14020506/s1. Supplementary Table S1. Whole genome sequence and analytical data of Salmonella isolates from children with SCD.

## Abbreviations

The following abbreviations are used in this manuscript:

ARG	Antibiotic Resistance Gene
iNTS	Invasive Nontyphoidal *Salmonella*
LMIC	Low- and Middle-Income Countries
MDR	Multidrug resistant
MGT	Multilevel Genome Typing
SCD	Sickle-Cell Disease
SPI	*Salmonella* Pathogenicity Island
sSA	Sub-Saharan Africa
ST	Sequence Type
WGS	Whole-Genome Sequencing
